# Can a Peer Support the Process of Self-Management in Narcolepsy? A Qualitative Narrative Analysis of a Narcoleptic Patient

**DOI:** 10.3389/fpsyg.2020.01353

**Published:** 2020-07-07

**Authors:** Christian Franceschini, Chiara Fante, Marco Filardi, Maria Claudia Folli, Francesca Brazzi, Fabio Pizza, Anita D’Anselmo, Francesca Ingravallo, Elena Antelmi, Giuseppe Plazzi

**Affiliations:** ^1^Department of Medicine and Surgery, University of Parma, Parma, Italy; ^2^Istituto per le Tecnologie Didattiche (ITD), National Research Council (CNR), Genova, Italy; ^3^Department of Biomedical and Neuromotor Sciences, Alma Mater Studiorum, University of Bologna, Bologna, Italy; ^4^IRCCS Institute of Neurological Sciences, Bologna, Italy; ^5^Department of Medical and Surgical Sciences (DIMEC), University of Bologna, Bologna, Italy; ^6^Neurology Unit, Movement Disorders Division, Department of Neurosciences, Biomedicine and Movement Sciences, University of Verona, Verona, Italy

**Keywords:** narcolepsy, case report, clinical psychology, peer support, self-management, coping strategies, narrative medicine

## Abstract

**Introduction:**

Narcolepsy type 1 (NT1) is a chronic and rare sleep disorder typically arising during adolescence and young adulthood. The main symptoms are excessive daytime sleepiness and cataplexy, a prototypical fall down elicited by huge emotions. Social relationships, school, work, and general health perception are frequently impaired in patients, who often show lower quality-of-life scores. We report which management strategies a young patient (DMG) adopted to cope with NT1 during his growth, avoiding exhibiting serious impairments to his global functioning.

**Methods:**

A clinical psychologist explores the history of the patient’s disease and the self-acquired strategies used to cope with the symptoms. The patient’s global adaptation to the disease, stress-related managing skills, and overall well-being are assessed by standardized scales [Illness Behavior Questionnaire (IBQ); Coping Orientations to Problems Experienced (COPE); and Psychological General Well-Being Index (PGWBI)]. We conducted a qualitative analysis of the patient’s narration of his illness according to the procedure of the Grounded Theory. The MAXQDA software program was used to code the verbatim transcript.

**Results:**

From the qualitative analysis of the interview, three thematic cores emerged: 1) the disease history; 2) the patient’s friendship with AD, a friend of his age diagnosed with NT1 since childhood; 3) the strategies used to deal with his symptoms before the diagnosis of NT1 and the related treatment. From the psychometric tests, the patient presents good coping strategies in dealing with stressful problems and events based mainly on acceptance and positive reinterpretation of the stressful situation.

**Conclusion:**

This case shows that comparing peers of the same age and suffering from the same illness improve the patient’s self-management ability to cope and live well with NT1.

## Introduction

Narcolepsy type 1 (NT1) is a rare and chronic central nervous system hypersomnia characterized by excessive daytime sleepiness (EDS), sudden loss of muscle tone elicited by intense emotions, usually laughing and joking (cataplexy), sleep paralysis, hypnagogic hallucinations, and disrupted nocturnal sleep [American Academy of Sleep Medicine (AASM), 2014]. The diagnosis relies on polysomnography (PSG) and multiple sleep latency test (MSLT) [American Academy of Sleep Medicine (AASM), 2014], documenting the neurophysiological hallmarks of narcolepsy: shortened sleep latency and sleep-onset rapid eye movement (REM) periods (SOREMPs). Low cerebrospinal fluid hypocretin-1 (CSF hcrt-1) levels (below 110 pg/ml) and EDS complaints are also sufficient to establish this diagnosis.

The disease is caused by hypocretin neuronal loss, most likely on autoimmune basis (Latorre et al., 2018).

NT1 onset displays a bimodal incidence peak around 15 and around 36 years of age (Plazzi et al., 2011; Thorpy and Krieger, 2014), with a prevalence of 0.02–0.06% in the American and European population (Mignot, 1998).

In NT1 patients, high comorbidity for other medical conditions is usually reported: weight gain up to obesity (Ponziani et al., 2016), precocious puberty (Postiglione et al., 2018), and psychiatric disorders, in particular depression (37.9%) (Dauvilliers et al., 2009; Fortuyn et al., 2011; Ruoff et al., 2017) and anxiety disorders (53%) (Fortuyn et al., 2010). An association with schizophrenia-like psychosis can also be noticed (Plazzi et al., 2015). Besides, from a psychological point of view, illness-related problems at school/work and in maintaining stable romantic relationships can seriously impair the patient’s quality of life (Ingravallo et al., 2012; Plazzi et al., 2018; Raggi et al., 2019).

Available treatments for NT1 are only symptomatic, primarily focused on EDS and cataplexy. The former is generally treated with stimulants, while antidepressants are suggested for cataplexy. Sodium oxybate (gamma-hydroxybutyric acid B-subtype receptor agonist - GABAb) and pitolisant (H3 receptor antagonist/inverse agonist) can be administered for both symptoms (Franceschini et al., 2020).

As we mentioned before, NT1 implies psychological issues that, if managed, can facilitate better adherence to pharmacological therapy and improve the quality of life. In this regard, as suggested by clinical international guidelines (Britton et al., 2002; Billiard et al., 2006), scheduling naps, work planning, psychosocial support, and behavioral therapy may reduce the negative consequences of this disease.

Quality of life in patients suffering from chronic disease is related to a good management of the disease. Accordingly, identifying and proposing novel management strategies to patients may significantly improve their overall well-being: psychological counseling successfully helps both the patient and his/her parents in developing management skills, and educational programs can improve the patient’s knowledge and confidence related to his/her disorder (Shilling et al., 2013).

Other techniques concentrate on recognizing symptoms and taking appropriate actions by developing strategies to deal with the illness and interact with the healthcare system over time (Schulman-Green et al., 2012). Among these, self-management is “*the ability of the individual, in conjunction with family, community, and healthcare professionals, to manage symptoms, treatments, lifestyle changes, and psychosocial, cultural, and spiritual consequences of health conditions*” (Richard and Shea, 2011) and stands as a dynamic and interactive process that directs individuals to manage a chronic illness (Lorig and Holman, 2003).

We present a case of a young NT1 patient who was able to develop the self-management process on his symptoms and perform academic activities without serious impairments. For this purpose, we conducted a qualitative analysis of the patient’s narration of his illness and correlated it to a psychological evaluation (carried out through standardized tests) to describe the management strategy/process that the patient adopted to cope with the symptoms.

The report has been conducted according to the CARE guidelines for case reports (Riley et al., 2017).

## Presenting Concern and Clinical Findings

At the age of 21, the patient (DMG are his fake initials) was referred to the center for narcolepsy of the University of Bologna for complaints of EDS and cataplexy.

During the clinical interview, the patient claimed to be already aware of having narcolepsy, as one of his classmates (AD are his fake initials) has been afflicted with the pathology since childhood. DMG recollected the onset of daytime sleepiness at the age of 6, especially in the early afternoon, with multiple sleep episodes when he was mentally exhausted or bored. The sleep need was described as unavoidable, and nap duration was generally short (i.e., 5 min) and associated with feelings of fatigue and a sensation of warmth upon awakening. The patient reported cataplexy episodes involving mainly the facial district and elicited by intense laugh (1–2 times a week). He never experienced sleep paralysis or hypnagogic/hypnopompic hallucinations.

At diagnostic hospitalization, the patient presented with severe sleepiness [Epworth Sleepiness Scale (ESS) = 18] (Johns, 1991; Vignatelli et al., 2003) and was markedly overweight [body mass index (BMI) = 29] (Ponziani et al., 2016).

**FIGURE 1:**
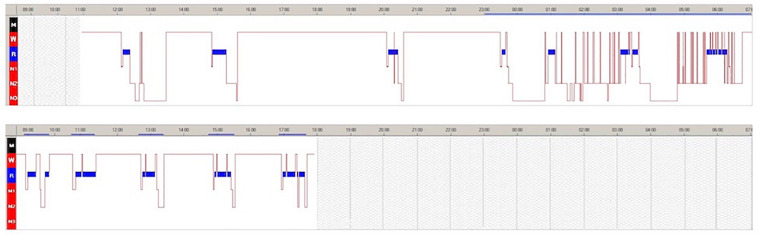
Twenty-four-hour video-polysomnography (v-PSG) **(A)** and multiple sleep latency test (MSLT) **(B)** during the hospitalizations. Blue bar indicates rapid eye movement (REM) sleep. The solid blue line indicates the period between when the light was switched off and when it was switched on.

The 24-hour continuous video-polysomnography (v-PSG) documented several daytime naps with SOREMPs. Nocturnal sleep was disturbed by frequent awakenings and described as non-refreshing (Figure 1A). The MSLT confirmed pathological sleep propensity (mean sleep latency = 2.30 min) with 5/5 SOREMPs (Figure 1B).

The patient had undetectable CSF hcrt-1. Accordingly, NT1 was diagnosed, and a treatment with sodium oxybate up to 9 g was started with a remarkable improvement on outpatient follow-up (after 12 months) on both hypersomnolence (ESS = 5) and cataplexy (1/2 times a month to the face); moreover, his weight returned within the normal range (BMI 25 kg/m^2^).

Although narcolepsy-related symptoms emerged in his clinical history since childhood, with an escalation during adolescence, by the time of the diagnosis, DMG showed quite a good adaptation. Indeed, as reported, the patient, already aware of the pathology’s symptoms for some considerable time, managed to deal with both sleepiness symptoms and cataplexy episodes, without falling into scholastic failures and/or social isolation.

## Material

An interview that lasted about an hour and a half with a clinical psychologist (ChrF) was carried out to collect the patient’s disease history and information on the self-acquired strategies adopted to cope with the symptoms. Moreover, the patient completed standardized scales assessing global adaptation to the disease, stress-related managing skills, and overall well-being. More in detail, the Coping Orientations to Problems Experienced (COPE) (Carver et al., 1989; Sica et al., 1997) was used to assess coping skills defined as the abilities and strategies used to deal with problematic or stress-related situations. The COPE consists of 15 scales (four items each), namely, *Activity, Planning, Suppression of competitive activities, Containment, Information research, Search for understanding, Emotional outburst, Positive reinterpretation and growth, Acceptance, Religious devotion, Humor, Mental detachment, Behavioral detachment, Denial*, and *Alcohol and drug use.* The Illness Behavior Questionnaire (IBQ) (Pilowsky and Spence, 1975; Fava et al., 1982) is used to evaluate modalities in which individuals react to aspects of their physical functionality and consists of 62 items that explore emotions and inclinations regarding the disease, perception of significant people’s reaction (including the physician), and vision of his psychosocial situation. The IBQ is formed by the following scales: *Global hypochondria, Belief to suffer from the illness, Psychological/somatic perception, Emotional inhibition, Denial*, and *Irritability*.

Finally, health-related quality of life was evaluated through the Psychological General Well-Being Index (PGWBI) (Dupuy, 1984; Grossi et al., 2002). The questionnaire consists of 22 items that explore six different dimensions: *Anxiety, Depression, Positivity and wellness, Self-control, All- around health*, and *Vitality.*

## Method

### Description of the Procedure

The Grounded Theory (GT) is a method inspired by an interpretative model that aims to deeply analyze the meanings connected to the studied subject (Arcidiacono and Gregorio, 2008). While a quantitative method aims to validate prior theories by referring to a linear paradigm, GT systematically gathers and analyzes research data that cannot be manipulated by the researcher but are instead coherent with the real collection situations. In this way, it is possible to generate theories truly based on data (Glaser and Strauss, 2017).

Accordingly, the open-codification procedure of the GT was followed in order to analyze the interview (Strauss and Corbin, 1990).

The recording of the interview was transcribed verbatim. The transcript was coded using the MAXQDA software program (VERBI GmbH, Marburg, Germany), applying open and axial coding (Boeije, 2010).

### Description of Data Analysis

We started with an explorative analysis of the general content, following a bottom-up approach where no categories are defined before looking at the material. To reduce errors in this process and identify all possible categories, two different researchers analyzed the material independently (ChrF; CF). As the study concerns a single case, we preferred to consider every single theme that emerged, without considering potential long passages.

Each of the researchers was asked to identify *in vivo* a series of emerging themes and possible subthemes (“codes”). A codebook was created based on the preliminary results achieved by the two encoders. Later, the two researchers (separately) analyzed the interview by assigning codes (identified according to their content) to specific portions of the text. This way, they obtained a satisfactory intersubjective agreement grade (kappa = 0.82).

Unrefined scores of each scale were calculated for every qualitative instrument that was administered to the patient and then converted into corresponding *Z*-scores.

## Results

### Standardized Tests

Results of standardized tests are reported in Table 1. Regarding coping strategies developed to deal with stressful situations, the patient shows significant marks in relation to the dimensions “Activity” and “Positive attitude.” These scales are considered adaptive in problem-focused coping: undertaking an action to eliminate or reduce stress effects (Activity scale, *z* = 1.5); thinking, planning and elaborating strategies to solve the problem (Planning scale, *z* = 1.5), and putting aside every other activity, avoiding distractions to better face situations (Suppression of competitive activities scale, *z* = 1.8) are modalities frequently used by the patient. Other significant strategies are critical elaboration (positive/growth) of his experience (Positive reinterpretation scale, *z* = 1.8) and acceptance of the situation and/or his inability to face it (Acceptance scale, *z* = 1.9). There is no evidence of an excessive resort to unsuitable modalities shaped on avoidance or denial (his marks do not deviate from the cross-section’s ones).

**TABLE 1:** Results collected from the patient’s responses to standardized tests.

**Coping Orientations to Problems Experienced (COPE)**	**Unrefined score**	***Z* – score**
Activity	14	1.4865
Planning	16	1.4961
Suppression of competitive activities	14	1.8426
Containment	13	1.3077
Information research	8	−0.393
Search for understanding	5	−1.19
Emotional outburst	8	−0.153
Positive reinterpretation and growth	16	1.7607
Acceptance	15	1.9084
Religious devotion	4	−0.947
Humor	9	0.4733
Denial	6	0.3801
Behavioral detachment	5	−0.369
Mental detachment	11	0.9816
Alcohol and drug use	4	−0.403
**Illness Behavior Questionnaire (IBQ)**	**Unrefined score**	**Hospitalized population**
Hypochondria	0	5.69
Belief to suffer from the illness	2	4.2
Psychological/somatic perception	2	2.73
Emotional inhibition	2	4.04
Dysphoria	0	4.45
Denial	1	3.9
Irritability	1	3.86
**Psychological General Well Being Index (PGWBI)**	**Unrefined score**	**50% Percentile Male, age 21–24**
Anxiety	20	18
Depression	10	14
Positivity	10	15
Self-control	10	13
Global welfare	5	14
Vitality	15	15
Total	70	88

The patient does not show any higher score on IBQ scales, suggesting the absence of clinically significant behaviors or attitudes related to the pathology (i.e., hypochondria, denial, or emotional inhibition). General perceived well-being, assessed with the PGWBI, was slightly lower than the reference value for the male population (70 vs. 88).

### Interview Analysis

Three thematic cores emerged from the qualitative analysis of the interview: (1) history of the disease; (2) story of DMG’s friendship with AD; (3) strategies (partially self-acquired) adopted to deal with the symptoms before diagnosis of NT1 and related treatment.

A series of codes were identified to describe each thematic core. The identified codes, their definition, and the number of text’s portions they codify are shown in Table 2.

**TABLE 2:** Identified codes for each thematic core, their definition and number of text’s sections codified.

Code	Definition	Number of text’s portions codified
**THEMATIC CORE: relationship with the peer afflicted with narcolepsy**		
*Knowledge of the disease thanks to his friendship with A.D.*	In this section, the patient claims he discovered and got familiar with the illness and related symptoms by observing and getting closer to A.D.	9
Awareness of the disease thanks to his friendship with A.D.	In this section, the patient declares he became aware of being afflicted with narcolepsy thanks to his friendship with his narcoleptic peer.	8
**THEMATIC CORE: strategies to cope with the disease**		
*Study planning through learning strategies*	In this section, the patient claims to cope with disease-related study limitations by using specific learning strategies	6
*Observation of peers*	In this section, the patient claims to cope with his symptoms by observing how the peer group face everyday life situations	3
*Stress management*	In this section, the patient copes with his symptoms by trying to properly manage everyday stress	1
*Sharing with peers*	In this section, the patient claims to cope with his disease by sharing experiences with his peers	7
*Management through physical activity*	In this section, the patient claims to cope with his disease by regular physical activity	2
*Management through beneficial sleep*	In this section, the patient claims to cope with his symptoms by planning and regulating his night-time sleep	2
*Management through naps*	In this section, the patient claims to cope with his symptoms by planning some naps during the day	7
*Problem-focused coping*	In this section, the patient describes situations where a problem-focused coping strategy is used	3
**THEMATIC CORE: history of the disease**		
*Initial awareness of the disease by identifying sleepiness*	In this section, the patient illustrates moments and episodes when he recognized the excessive sleepiness as a symptom	3
*Initial awareness of the disease by identifying cataplexy-related symptoms*	In this section, the patient illustrates moments and episodes when he recognized muscular failure as a symptom	7
*Early memories of cataplexy-related symptoms*	In this section, the patient explains cataplexy-related symptoms that affected his life	4
*Early memories of sleepiness*	In this section, the patient illustrates his life-time memories of sleepiness	6
*Mother’s memories of symptoms during childhood*	In this section, the patient claims that his mother narrates about narcolepsy-related symptoms dating back to childhood	3

Within the “History of the disease” thematic core, we collect the patient’s early memories originated from what his mother told him about his childhood (code: “*mother’s memories of symptoms during childhood*”), together with DMG’s own memories about EDS (code: “*early memories of sleepiness*”) and cataplexy episodes (code: “*early memories of cataplexy-related symptoms*”) during his adolescence.

With regard to the “Relationship with his peer afflicted with narcolepsy,” in the patient’s report, we notice the importance of his relationship with his friend of the same age afflicted by NT1. The comparison between his situation and the one of his peer allowed the patient to better recognize NT1 symptoms (code: “*knowledge of the disease thanks to his friendship with AD*”) but also raise his awareness about it (code: “*awareness of the disease thanks to his friendship with AD*”).

Finally, in the thematic core, “Strategies to cope with the disease,” some strategies used by the patient to manage his condition, implemented before having a confirmed diagnosis, emerged. They vary from management of sleepiness symptoms (code: “*management through naps*” and “*management through good sleep*”) to specific learning strategies to fulfill academic requests and reduce limitations (code: “*study planning through learning strategies*”) and global adaptive stress management strategies (code: “*problem-focused coping*,” “*stress management*,” and “*observation of peers*”). Moreover, DMG reported a regular practice of physical activity as a means to maintain his well-being (code: “*management through physical activity*”).

Example sentences for each thematic core and codes from the patient’s interview are reported in Table 3.

**TABLE 3:** Example sentences for each thematic core and codes from the patient interview.

Code	Examples
**THEMATIC CORE: relationship with the peer afflicted with narcolepsy**	
*Knowledge of the disease thanks to his friendship with A.D.*	*“I’ve known A.D. since I was a child, so I knew he was narcoleptic*… *everybody could see it because he used to fall asleep in class all at once” “*…*for example, in middle school we used to hang out together on Saturday nights. One day we spent the night by him and he took his pills the morning after, so that night I asked him what they were for, even if I already knew. I knew that by then his symptoms showed up only during the day, so he explained me*…*”*
*Awareness of the disease thanks to his friendship with A.D.*	*“*…*I was quite well aware of my condition when I was in my last year of high school. In a sort of way, I began to compare myself to A.D., who I was sure was narcoleptic*…*” “*…*I realized that our conditions were very similar, maybe his symptoms were a little more serious than mine, in particular compared to cataplexy, but more or less it was the same thing*… *I often fell asleep.” “I started thinking about A.D. because he usually takes a nap after lunch in order to be awake when we’re together, otherwise he would fall asleep. And I began to think that I use to fell asleep the very same way indeed.”*
**THEMATIC CORE: strategies to cope with the disease**	
*Study planning through learning strategies*	*“Well*… *yes. I constantly had to rework the best way to follow my lessons because when I used to write them in pencil I often found myself with missing parts that I couldn’t remember anymore. Then I started to write in pen (even if I’m not good with handwriting) and to underline concepts in my book as soon as the teacher explained them. This way when I get home I can immediately see everything I need to learn at a glance. I noticed that planning my study has worked.” “So, comparing myself to them, observing their study method, their interest*… *I started to understand that I needed that interest in things too. And that I needed that extra time as well.”*
*Observation of peers*	*“We study together or we take a walk sometimes*… *he’s really good because he’s full of interests. He has been taught not to always want to be the best, not to be that strict with school and to develop whatever kind of thing he liked. So, comparing myself with him I learned a lot myself.”*
*Stress management*	*“Yet I realized that even if I stay at home and study on Saturdays, the situation doesn’t change that much*… *it’s better to set the day free from study and stress, which helps.”*
*Management through physical activity*	*“Indeed, I used to run 3 km every day and do a bunch of exercises before my hospitalization. I feel better with a healthy breakfast.”*
*Management through beneficial sleep*	*“To be awake at 6 I need to be in bed at 11, so I force myself not to stay up longer than midnight. This way I control myself and sleep for at least 7 h.”*
*Management through naps*	*“*…*Now I can basically figure out when to sleep, such as during breaks. It’s perfect, I sleep in those 15 min when there’s no professor in class*…*” “I start to plan my daytime because indeed it’s the only thing that works for me. I know that the nap works.”*
*Problem-focused coping*	*“I’ve always tried to do all of my exams as fast as I could, to finish as soon as possible. I tried to find a compromise now that I’m in college: I want to pass my exams before the end of this academic year, but I want to pass them good, so I’m trying to split them. I don’t want to overload myself because I know I won’t be good at handling them.” “Now I’m beginning to talk about it (the disease) when I’m with my friends, actually it’s pretty obvious*…*”*
**THEMATIC CORE: history of the disease**	
*Mother’s memories of symptoms during childhood*	*“*… *My mother always tells about how much I used to sleep in the afternoon, sometimes in an irregular way*… *I mean, not an after-lunch nap, but also later, before dinner.”*
*Early memories of sleepiness*	*“I don’t have any memories of primary school, but I remember that in high school I started to fall asleep in class and I wasn’t able to follow the entire lesson throughout.” “I became aware that this happened to me most during the high school period*… *from first to last year I became more and more conscious of my falling asleep after certain events.”*
*Early memories of cataplexy-related symptoms*	“Sometimes I could fall down. For example, I remember that when I used to act like a nut or when I used to jest with my parents, joking after lunch because I was happy, or I had got a good grade or something like that… it happened to me to laugh out loud, also having to sit down because my legs couldn’t bear me.” *“I realized to be narcoleptic around my last high school year because of this sense of feeling faint when I was laughing, so basically because of cataplexy rather than drowsiness.”*

## Discussion

This is the first qualitative study focused on symptomatology self-management in a patient with NT1, a potential approach to facilitate well-being and coping strategies in patients affected by this sleep disease.

Interestingly, before the diagnosis, the patient, although untreated and without any medical advice, showed an optimal adaptation to narcolepsy symptoms. Standardized tests did not highlight any particular critical areas, except for moderate levels of general distress, confirming proper psychological and social functioning. Regarding his disease history, no unusual and suffering-orientated behaviors have been observed. Moreover, DMG adopted good coping strategies, based on acceptance and positive reinterpretation of situations, to face problems and stressful events.

Qualitative analysis of the patient seems to confirm the collection of these heterogeneous symptoms in the diagnosis of NT1. DMG is aware of the disease, thanks to his relationship with a peer diagnosed with NT1 during childhood. This friendship allowed DMG to become familiar with his symptoms and recognize them in his global functioning (code: “*knowledge of the disease thanks to his friendship with AD*”): the fact that DMG attributed his first episodes of muscular weakness and sudden sleep attacks to a medical condition (code: “*awareness of the disease thanks to his friendship with AD*”) stands as a striking example of such process.

The opportunity to compare what he was undergoing with the experience of his friend provided him an explanation that has reasonably reduced his feelings of insecurity, confusion, or fear, which are typical of NT1 patients before getting a diagnosis (Thorpy and Krieger, 2014). Besides, the patient was able to acquire functional strategies to manage NT1 symptoms, especially sleepiness (taking several short naps). Accordingly, the patient’s statements confirm the hypothesis that peers’ support and mutual learning are key factors to improve management skills and self-trust and therefore reduce the sense of diversity and contribute to a more positive self-image (Johnston et al., 2012; Jormfeldt et al., 2012).

The currently available literature proposes a distinction in three macro-categories when referring to self-management processes related to chronic illnesses (Schulman-Green et al., 2012): (a) focusing on illness needs (i.e., all tasks and skills needed to the individual to manage daily needs); (b) activating resources (i.e., all individuals and social resources); and (c) living with a chronic illness (i.e., all tasks and skills related to coping with the illness and growing as a person). By analyzing the patient’s interview, we can see how he developed adaptive strategies for each of these categories; for instance, as for “Focusing on illness needs (a),” the patient reports how he learned to reduce sleepiness symptoms by adequate sleep hygiene, planned naps throughout the day, and physical activity. DMG seemed to have globally activated psychological and social resources, and therefore, he managed his most stressful moments by sharing with peers and learning through observation (b). Finally, organizing specific strategies for studying and a problem-oriented coping style allowed him to live well with NT1 (c). COPE results coupled with the evaluation of the qualitative interview analysis show how the patient tends to activate confident strategies like thinking, planning, and elaborating ways to overcome problems when faced with stressful situations, which are re-elaborated in a positive connotation.

Moreover, literature has often reported that among all symptoms, EDS has a major impact on the quality of life, relationships, and school/work conditions of an NT1 patient: being in public may be extremely troubling because of the fear of a sudden sleep attack or falling to the ground as a consequence of cataplexy (Daniels et al., 2001; Vignatelli et al., 2004, 2011; Avis et al., 2015; Raggi et al., 2019). This constant preoccupation with others’ opinions often results in a stigmatization process that affects these patients’ social life (Rovere et al., 2008; Kapella et al., 2015; Franceschini et al., 2020). It has been clear for years (Alaia, 1992; Rovere et al., 2008; Franceschini et al., 2020) that narcolepsy treatments cannot be limited to pharmacological therapy but needs to be implemented with social support and help from a peer who is aware of the challenges brought by this disease.

Finally, this clinical observation of DMG’s story supports the hypothesis that patients with chronic illnesses can feel better and generally more hopeful about their condition when they can compare themselves to someone else who, suffering from the same disease, seems to manage his/her symptoms efficiently (upward social comparison) (Stenberg et al., 2016). One of the key strengths of this manuscript is that it offers the opportunity to develop a discussion around innovative approaches in increasing NT1 patients’ management skills and adaptation. Providing settings where patients can freely share their everyday problems and experiences may ease the process of dealing with symptoms-related emotions, as well as discovering new self-management strategies and finally gain a more fulfilling quality of life. On the other hand, a possible limitation of this report may be the fact that it reflects only the psychological experience and outcomes of a single individual. Nevertheless, this study certainly underlines the importance of creating a setting where NT1 patients can get a real feeling of understanding when explaining controversial emotions, such as peer support-oriented groups and psycho-educational interventions to promote adaptation and management skills. Finally, future clinical research studies should be centered on understanding the role played by patients’ coping strategies and by familial environmental factors to better help the well-being of NT patients.

## Conclusion

To date, this is the first original study adopting a qualitative approach to explore the efficacy of peer support in self-management processes specific for NT1. The clinical history of DMG and his management strategies, strongly influenced by the observation of his friend afflicted with the same illness, suggest the importance for patients to share their own experiences. Creating settings where NT1 patients can interact with each other and talking about symptoms may be strongly beneficial, together with providing support for those who are experiencing NT1 symptoms for the first time.

The opportunity to learn new perspectives about perceiving and managing the disease represents a crucial element of an effective self-management promoting team for patients with NT1.

## Ethics Statement

Ethical approval was not provided for this study on human participants because the study has been conducted according to the principles set forth by the Declaration of Helsinki (59th WMA General Assembly, Seoul, October 2008) and in accordance with the Medical Research Involving Human Subjects Act (WMO). Written informed consent was obtained from DMG both for the purposes of research participation as well as for the publication of the case report, including indirectly identifiable data. The patients or participants provided their written informed consent to participate in this study.

## Author Contributions

The case report study was based on a concept developed by CFr who wrote the manuscript and took part in the review and critique processes as PI. CFr conducted the clinical interview to the patient. CFa organized the study, performed the neuropsychological assessment (organization and execution), and participated in the review and critique processes. MF participated in the interpretation of all the psychological results and participated in the review and critique processes. MCF participated in the review and critique process of manuscripts. FB, FP, FI, and EA critically reviewed the manuscripts and gave their approval of this version of the manuscript to be submitted. GP had followed the clinical developmental of the case report and the critical review process of manuscripts. All authors contributed to the article and approved the submitted version.

## Conflict of Interest

GP participated in the advisory board of UCB Pharma, Jazz Pharmaceuticals, and BioProject. The remaining authors declare that the research was conducted in the absence of any commercial or financial relationships that could be construed as a potential conflict of interest.

## Abbreviations

EDS: excessive daytime slepiness
NT1: narcolepsy type 1.
